# Reasons to avoid vitamin D deficiency during COVID-19 pandemic

**DOI:** 10.20945/2359-3997000000291

**Published:** 2020-08-24

**Authors:** Rodrigo Nolasco dos Santos, Sergio Setsuo Maeda, José Roberto Jardim, Marise Lazaretti-Castro

**Affiliations:** 1 Universidade Federal de São Paulo Departamento de Medicina Disciplina de Endocrinologia São Paulo SP Brasil Disciplina de Endocrinologia, Departamento de Medicina, Universidade Federal de São Paulo (Unifesp), São Paulo, SP, Brasil; 2 Universidade Federal de São Paulo Departamento de Medicina Disciplina de Pneumologia São Paulo SP Brasil Disciplina de Pneumologia, Departamento de Medicina, Universidade Federal de São Paulo (Unifesp), São Paulo, SP, Brasil

**Keywords:** Coronavirus, vitamin D, respiratory infections, immune system

## Abstract

The effects of vitamin D on the musculoskeletal system are well established. Its deficiency causes osteomalacia, secondary hyperparathyroidism, and an increased risk for fractures and falls. However, clinical and experimental evidence points to extra-skeletal actions of vitamin D, including on immune and respiratory systems. Thus, during this COVID-19 pandemic, a possible deleterious role of vitamin D deficiency has been questioned. This paper aims to present a brief review of the literature and discuss, based on evidence, the role of vitamin D in the lung function and in the prevention of respiratory infections. Relevant articles were searched in the databases MEDLINE/PubMed and SciELO/LILACS. The mechanisms of vitamin D action in the immune system response will be discussed. Clinical data from systematic reviews and meta-analyses show benefits in the prevention of respiratory infections and improvement of pulmonary function when vitamin D-deficient patients are supplemented. At the time of writing this paper, no published data on vitamin D supplementation for patients with COVID-19 have been found. Vitamin D supplementation is recommended during this period of social isolation to avoid any deficiency, especially in the context of bone outcomes, aiming to achieve normal values of 25(OH)D. The prevention of respiratory infections and improvement of pulmonary function are additional benefits observed when vitamin D deficiency is treated. Thus far, any protective effect of vitamin D specifically against severe COVID-19 remains unclear. We also emphasize avoiding bolus or extremely high doses of vitamin D, which can increase the risk of intoxication without evidence of benefits.

## INTRODUCTION

In December 2019, an outbreak of pneumonia of unknown origin was initially reported in Wuhan, Hubei Province, China. SARS-CoV-2 (previously 2019-nCoV), a single-stranded RNA virus, was identified as the agent. Six other kinds of coronaviruses are known to cause human disease, including severe acute respiratory syndrome coronavirus (SARS-CoV) and Middle East respiratory syndrome coronavirus (MERS-CoV), which caused outbreaks in 2003 and 2012, respectively. SARS-CoV-2 is highly similar to a bat coronavirus, indicating that bats can be the natural reservoir host of various SARS-related coronaviruses and could also be the original host of SARS-CoV-2. Coronavirus disease 2019 (COVID-19) was declared a global pandemic on March 11, 2020 (1-3).

According to the COVID-19 Dashboard by the Center for Systems Science and Engineering at Johns Hopkins University, on June 22, 2020, a total of 9 million cases and 469,122 deaths were reported around the world (4).

The median incubation period is 5.1 days, and 97.5% of the infected subjects will develop symptoms within 11.5 days of infection. Fever (88.5%), cough (68.6%), myalgia or fatigue (35.8%), expectoration (28.2%), and dyspnea (21.9%) are the most common symptoms reported (2). There is a wide range of clinical manifestations in patients with SARS-CoV-2, from mild, to moderate, to severe and fulminant disease. In those patients who develop pneumonia, ground-glass opacity and multiple mottling are described on thorax computed tomography (2). In 10% to 20% of severe patients, the respiratory injury will develop into acute respiratory distress syndrome (ARDS) with high morbidity and mortality. Risk factors for developing severe to critical cases include advanced age and underlying comorbidities such as hypertension, diabetes, and cardiovascular disease (1).

Regarding the pathogenic mechanism that produces pneumonia, the available data suggest that the COVID infection is capable of producing an excessive immune reaction in the host (“cytokine storm”) and extensive tissue damage, especially in the lungs (5).

In general, innate immune responses (Toll-like receptors, type I interferons, macrophages, and dendritic cells) represent the initial host defense against invading pathogens. This inhibits virus replication, promotes virus clearance, induces tissue repair, and triggers a prolonged adaptive immune response (T-cells produce proinflammatory cytokines via the NF‐kB and MAPK signaling pathway) against the viruses. In most cases, pulmonary and systemic inflammatory responses associated with CoVs are triggered by the innate immune system when it recognizes the viruses (6,7).

The main factor of this storm in COVID-19 seems to be interleukin 6 (IL-6), which is produced by activated leukocytes (8). Giamarellos-Bourboulis and cols. described a pattern of immune profile in severe cases characterized by IL-6-mediated low HLA-DR expression and lymphopenia, associated with sustained cytokine production and hyper-inflammation (9). Huang and cols. showed that intensive unit care patients had higher plasma levels of IL-2, IL-7, IL-10, GSCF, IP10, MCP1, MIP1A, and TNF-α (10).

Vitamin D has been investigated for its immunomodulatory action related especially to respiratory infections. The discussion proves to be quite timely in the face of the coronavirus pandemic.

## VITAMIN D

**Figure 1:**
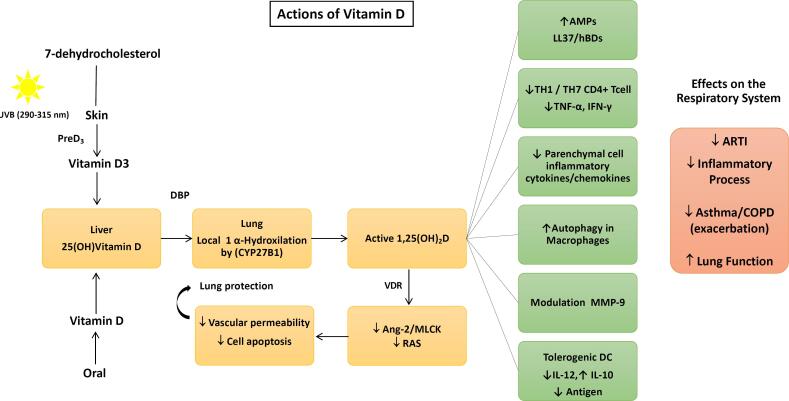
Proposed mechanism whereby 1,25(OH)_2_D_3_ -VDR signaling acts on the respiratory system (adapted according to references 7, 13, 14, and 29. Abbreviations: DBP = vitamin D binding protein; Ang-2 = angiopoietin-2; MLCK = Ang-2-Tie-2-kinase cascade – MLC = myosin light chain; RAS = renin-angiotensin system; DC = dendritic cell; BC = B-cell (B cell: B lymphocyte, cyto T cell, cytotoxic T cell); VDR = vitamin D receptor; T cell = T lymphocyte; TH = helper T cell; Treg = regulatory T cell; IL = interleukin; TNF = tumor necrosis factor; INF = interferon; AMPs = antimicrobial peptides cathelicidin (LL-37) and human beta-defensins (hBDs), and MMP-9 = matrix metalloproteinase 9; ARTI = acute respiratory tract infection; COPD = chronic obstructive pulmonary disease.

Although it is called a vitamin, vitamin D is conceptually a pre-hormone that, together with parathyroid hormone (PTH), acts as an important regulator of calcium homeostasis and bone metabolism. It can be obtained from food sources, but endogenous skin synthesis triggered by ultraviolet radiation type B is the main source. The vitamin D produced in the skin or ingested as a nutrient or supplement is transported into the liver, where it is converted into 25-hydroxyvitamin D [25(OH)D], or calcidiol, which is the main form of circulating vitamin D and also used for serum dosage, though still not active. In the kidney, 25(OH)D is converted to its biologically active form, 1,25(OH)_2_D_3_, or calcitriol through the enzyme 1α-hydroxylase. The main actions of 1,25(OH)_2_D_3_ are mediated by the nuclear transcription factor, called the vitamin D receptor (VDR), located in the nucleus of the cells (11) (Figure 1). The classic actions of vitamin D are related to bone metabolism, where it modulates PTH synthesis, promotes calcium absorption by the intestine, and in the kidneys, the 1,25(OH)_2_D stimulates calcium reabsorption of glomerular filtrate, and these actions are related to better bone mass and muscle function (11). In addition, vitamin D receptors and the enzyme 1α-hydroxylase have been found in cells of the immune system such as neutrophils, macrophages and dendritic cells, which reinforces the hypothesis of vitamin D effects beyond the musculoskeletal system (12).

Furthermore, an experimental research demonstrated that calcitriol modulates the expression of the angiotensin-converting enzyme 2, which is the host receptor for SARS-CoV-2 entry into cells. VDR-null mice presented more severe acute lung injury in a LSP-induced sepsis model, compared with wild-type counterparts. Likewise, calcitriol acts in the regulation of phosphorylation of myosin light chains, the main indicator of vascular permeability. Thus, as a result of the modulation of these systems, by which they act on vascular permeability, as well as the blocking of cell apoptosis in the lung, together, they might prevent the worsening of pulmonary disorders (13).

Concerning the non-skeletal actions of vitamin D, many systematic reviews and meta-analyses showed that its administration is associated with reductions in upper respiratory infections, exacerbation of asthma, and mortality, and these data will be discussed below (Table 1).

**Table 1:** Recent systematic reviews and meta-analysis from observational and randomized controlled studies evaluating vitamin D effects in immune system and respiratory infections

Author (ref.)	Type of study	N° of studies	N° of patients	Participants age	Main conclusions
Li, 2019 (41)	Observational	8	337 CRSP 179 CS	Not given	VDD association with CRSP, especially in patients with nasal polyps' patients.
Zhou, 2019 (42)	Observational	8	20,966	Not given	VDD patients experienced a significantly increased risk of CAP (OR = 1.64).
Pham, 2019 (43)	Observational	24	Risk of ARTI: 78,127; Severity of ARTI: 1,495	≥12 to 97 years	Higher risk of ARTI in the lowest 25(OH)D category. For each 4 ng/mL ↓ in 25(OH)D, the Odds of ARTI ↑ by 1.02. This was a non-linear trend, with ↑ in risk of ARTI occurring at 25(OH)D < 15 ng/mL)
Cariolou, 2019 (44)	Observational	52	7,434	0 to 18 years	The prevalence of VDD (<20 ng/mL) was 54.6%. Children with sepsis prevalence was 64.0% in those with RTI. ↑ risk of death in VDD children.
Gou, 2018 (45)	Observational	10	2,672	<18 years	TB was significantly associated with VDD in children. VD levels were significantly lower in TB patients than in controls
Huang, 2017 (46)	Observational	38	3,599 TB 3,063 CS	Not given	Association between VDs and TB; VDD such as risk factor for TB.
Jat, 2017 (47)	Observational	12	2,279	0 to 5 years	Children with LRTI were found to have significantly lower mean VD as compared to controls. There was likewise a correlation between VD levels and incidence and severity of LRTI.
Feng, 2017 (48)	Observational	16	Asthma: 8, 871; Wheeze: 9,072; RTI: 8, 359	0 to 9 years	~28% of participants had 25(OH)D of ≥30 ng/mL. Birth cohort studies appointed that ↑ in utero exposure to 25(OH) D is inversely associated with the risk of asthma and wheeze during childhood, demonstrating more than 20% ↓ risk.
Martineau, 2019 (29)	RCTs	25	11,321	0 to 95 years	VDS ↓ the risk of ARI among all participants. Effects of VDS were stronger in individuals with a baseline 25(OH)D < 10 ng/mL than in those with a baseline 25(OH)D of ≥ 10 ng/mL. Daily or weekly VDS, but not in those receiving one or more bolus doses experienced benefits.
Zhang, 2019 (49)	RCTs	5	1,126	≥18 years	VDS did not shorten the time to sputum culture and smear conversion and did not lead to an increase in the proportion of participants with negative sputum culture. However, it reduced the time to sputum culture conversion in the sub-group of participants with *Taq*/ tt genotype (HR 8.09) and improved the multidrug-resistant TB sputum culture conversion rate (RR 2.40).
Wu, 2018 (50)	RCTs	8	1,787	26.7 to 43.7 years	VDS ↑ the proportion of sputum smear and culture conversions, PCc, LC, and chest radiograph, but had no impact on adverse events and mortality. VDS can be considered as a combination therapy in PTB.
Yakoob, 2016 (51)	RCTs	4	3,198	<5 years	VDD did not demonstrate benefit on the incidence of pneumonia, diarrhea, death or RI in children.
Vuichard Gysin, 2016 (52)	RCTs	15	7,053	Median age: 19 years	73% of the participants that measured 25(OH)D were insufficient (≤20 ng/mL). In healthy individuals VDD does not reduce the risk of RTIs.

Control subjects (CS); randomized controlled trials (RCTs); decrease (↓); increase (↑); vitamin D status (VDs); serum 25-hydroxyvitamin D (25(OH)D); vitamin D deficiency (VDD); vitamin D supplementation (VDS); Odds ratio (OR); plasma calcium concentration (PCc); community acquired pneumonia (CAP); chronic rhinosinusitis patients (CRSP); lymphocyte count (LC); tuberculosis (TB); pulmonary tuberculosis (PTB); acute respiratory tract infection (ARTI); lower respiratory tract infections (LRTI) and respiratory tract infections (RTIs).

## VITAMIN D AND THE IMMUNE RESPONSE AGAINST RESPIRATORY INFECTIONS: EXPERIMENTAL DATA

VDR and vitamin D enzymes are present in virtually all cells of the innate and adaptive pathways of the immune system. These cells produce locally, and the expression of CYP27B1 is regulated by a network of immunoregulatory 1,25(OH)_2_D_3_ rather than calcium homeostatic inputs (14).

In vitro and experimental studies show that vitamin D enhances cellular innate immunity partly through the induction of antimicrobial peptides, including human cathelicidin LL-37, by 1,25(OH)_2_D_3_, and beta-defensins (7). Cathelicidins exhibit direct antimicrobial activities against a spectrum of microbes, including viruses. Vitamin D is also involved in cellular immunity, partially reducing the cytokine storm induced by the innate immune system. 1,25(OH)_2_D_3_ suppresses responses mediated by the T helper cell type 1 (Th1) by repressing the production of inflammatory cytokines IL-2 and interferon-gamma (INFγ). Additionally, 1,25(OH)_2_D_3_ promotes cytokine production by T helper type 2 (Th2) cells, which helps enhance the indirect suppression of Th1 cells by complementing this with actions mediated by a multitude of cell types (15-17) (Figure 1).

Immune and structural cells in the lung can express the 1α-hydroxylase enzyme CYP27B1, suggesting that localized synthesis of active vitamin D from circulating precursors could be an important mechanism for vitamin D to exert effects at sites of inflammation. Liu and cols. demonstrated that after an allergic stimulus, levels of cathelicidins, 1,25(OH)_2_D_3_, and 25(OH)D increased in bronchoalveolar lavage fluid (18).

## VITAMIN D AND THE IMMUNE RESPONSE AGAINST RESPIRATORY INFECTIONS: CLINICAL DATA

In the past, tuberculosis patients were sent to sanatoriums, where treatment included exposure to sunlight, which was thought to directly kill Mycobacterium tuberculosis. Curiously, in 1849, Williams described the use of cod liver oil in the treatment of tuberculosis, before the discovery of vitamin D (19). In general, infections are known to contribute to death in nursing home patients or those with a critical illness.

Recently, Ilie and cols. observed a negative correlation between mean 25(OH)D levels of the population from 20 European countries and COVID-19 mortality, as the number of people infected. Elderly people are the most vulnerable group for COVID-19 infection, as well as the group that has the most deficient vitamin D levels (20).

The Third National Health and Nutrition Examination Survey evaluated 18,883 participants older than 12 years in the US. After adjustments to the season of the year, body mass index, smoking history, asthma, and chronic obstructive pulmonary disease, the authors found that serum 25(OH)D levels had an independent, inverse association with a recent upper respiratory tract infection. Although 25(OH)D levels less than 30 ng/mL and these infections were more common in the winter season, 25(OH)D levels and respiratory infections were still inversely associated across all seasons (21).

In a blinded prospective cohort study, Sabetta and cols. evaluated monthly concentrations of 25(OH)D over the fall and winter 2009-2010 in 198 healthy adults. Concentrations of 38 ng/mL or more were associated with a significant two-fold reduction in the risk of developing acute respiratory tract infections and with a marked reduction in the percentages of days ill. Although this was an observational study, it is possible to speculate on whether reaching these levels with cholecalciferol administration would reduce the risk of acute respiratory infection (22).

No prospective studies have been published evaluating the benefits of vitamin D administration in COVID 19 patients so far. However, in HIV patients, vitamin D administration induced a potential reduction in secondary microbial infections such as tuberculosis, as well as an increase in CD4+ T-lymphocyte count and a decreased in biomarkers associated with chronic inflammation (23).

In a randomized placebo-controlled double-blind trial, 86 participants received 1,000 IU of oral vitamin D_3_ or placebo for 90 days. Vitamin D supplementation significantly increased airway surface liquid antimicrobial activity and serum concentration of 25(OH)D. Interestingly, in a subgroup analysis, Vargas Buonfiglio and cols. found that smokers did not have an increase in their baseline antimicrobial activity in response to vitamin D (24). In a post-hoc analysis from another large randomized, double-blind, placebo-controlled trial, vitamin D supplementation improved lung function demonstrated through the forced expiratory volume in 1 s (FEV1), but only among ever-smokers, and especially in those with vitamin D deficiency and with asthma and COPD (25).

In a longitudinal study, a cohort of healthy Mexican participants older than 55 years (n = 23) was followed for 12 months and evaluated every three months. Their 25(OH)D levels remained below 30 ng/mL throughout all seasons. Inflammatory markers such as IL-1β, IL-6, IL-10, IL-18, MCP-1, and TNF-α significantly varied with the season independently of 25(OH)D concentrations, considering age, sex, and individual as random effects, with the highest levels occurring during autumn and/or winter, and the lowest levels occurring in the spring. In this study, TNF-α levels were dependent on 25(OH)D concentrations, being higher in individuals with vitamin D deficiency (26).

Jolliffe and cols. published a systematic review of clinical studies (39 studies: 4 cross-sectional studies, 8 case-control studies, 13 cohort studies, and 14 clinical trials) evaluating the association between vitamin D deficiency and susceptibility to acute respiratory infection in humans. The observational studies reported statistically significant associations between low vitamin D status and increased risk of both upper and lower respiratory tract infections (27).

Table 1 summarizes the most recent meta-analyses evaluating observational and intervention data of vitamin D in the immune system and respiratory infections.

Rejnmark and cols. (28) published a systematic review of meta-analyses and randomized controlled trials (RCTs) focusing on the effects of vitamin D supplementation on the risk of respiratory tract infections. They observed that these studies were included relatively small samples and were of short duration. The beneficial effects of vitamin D supplementation were reported in some of the RCTs. In conclusion, the pooled overall findings suggest a beneficial effect of vitamin D on respiratory tract infections, despite it not being possible to discriminate the etiology of the infections, according to the authors. The studies included in this meta-analysis, however, were quite heterogeneous, including participants from newborns to elderly and with a wide range of infectious diseases, raising questions about the accuracy of these conclusions.

A more recent meta-analysis by Martineau and cols. (25 RCTs, 11,321 participants, aged from 0 to 95 years) showed that vitamin D supplementation reduced the risk of acute respiratory infections among all participants by 12% (aOR 0.88, 95%CI 0.81-0.96), and subgroup analysis revealed that protective effects were seen only in individuals receiving daily or weekly vitamin D without additional bolus doses (aOR 0.81, 95%CI 0.72-0.91). These protective effects of vitamin D were stronger in individuals taking daily or weekly doses together with a baseline 25(OH)D < 10 ng/mL (aOR 0.30, 95%CI 0.17-0.53) than in those with a baseline 25(OH)D ≥ 10 ng/mL (Table 1). Nonetheless, the authors warned that the causes of the infections in the study were diverse, and radiological confirmation was carried out in the minority of events (29).

Autier and cols. published recently a systematic review of meta-analyses and randomized trials on non-skeletal effects of vitamin D supplementation, which included 28 meta-analyses with participants of all ages who used cholecalciferol or ergocalciferol administered as oral or injectable preparations. The authors concluded that vitamin D supplementation is associated with a reduction in cancer mortality, especially in middle-aged and older people (doses of 800-1,200 IU per day of vitamin D), mainly when they are in the hospital or are institutionalized. However, the main finding highlighted by this study is that vitamin D supplementation might help to prevent common upper respiratory tract infections and asthma exacerbations, mostly in people with low 25(OH)D concentrations. The authors hypothesized that vitamin D supplementation could promote immunomodulatory effects that reinforce resistance to acute infections, which could reduce the mortality in debilitated individuals (30). On the other hand, this systematic review found no evidence that vitamin D supplementation affects biomarkers of systemic inflammation, and the authors suggested that low 25(OH)D could be a consequence of systemic inflammation rather than a cause (30).

Nevertheless, vitamin D plays a role in regulating human lung fibroblast functions in wound repair and tissue remodeling (31). In a post hoc analysis, vitamin D supplementation also improved spirometric parameters of lung function in postmenopausal women without regular practice of physical activity, compared with those not supplemented (32). Additionally, meta-analyses of trials in children, adults, and pregnant women showed that vitamin D supplementation can reduce the risk of exacerbations in patients with mild and moderate asthma who require corticosteroid treatment (33,34).

It is important to highlight that vitamin D deficiency is common mainly among the elderly, who are at risk for severe COVID 19 infection, especially during the period of lockdown, social distancing, and wintertime (11,35). Likewise, other patients who belong to the groups at risk for this infection are associated with low levels of vitamin D (Table 1). Lack of physical activity outdoors also favors less sun exposure (36). So far, there is no evidence that vitamin D protects specifically against SARS-CoV-2 infection, but it prevents respiratory infections and regulates the cytokine inflammatory response, possibly reducing the risk of subsequent ARDS (29,37).

The reasonable recommendation is to maintain levels of 25(OH)D above 30 ng/mL in the risk groups, including individuals **over 60 years old** (38).

## THE LACK OF EVIDENCE FOR THE RECOMMENDATION OF BOLUS OR HIGH DOSES VITAMIN D

Due to the worldwide repercussions of the coronavirus pandemic, there is an unbridled search for therapies to treat or minimize the effects of the COVID infection. In this context, some recommendations extrapolate data from other observations, suggesting the use of very high doses of vitamin D against infections (16).

Regarding the routes, oral or intramuscular, of vitamin D administration, in a prospective intervention study, was observed in elderly that oral 600,000 UI single dose of cholecalciferol or ergocalciferol showed to be more effective in increasing serum 25(OH)D than the equivalent intramuscular dose and is more rapidly metabolized. Although the authors had appointed greater efficacy of cholecalciferol than of ergocalciferol in improving vitamin D status regardless of the route of administration (39), clinical data suggest that daily or weekly doses offer better results than bolus (29) in the protection against acute pulmonary infections (Table 1). In addition, supplementation with extremely high doses of vitamin D could be harmful, especially to elderly individuals, and can potentially lead to falls and fractures (38,40).

Hypervitaminosis D increases intestinal calcium uptake, renal tubular reabsorption, and bone resorption, leading to hypercalcemia and related symptoms such as nausea, vomiting, weakness, anorexia, polydipsia, polyuria, dehydration, acute renal failure, apathy, confusion, and, in the long term, kidney stones. The recent consensus of the Brazilian Society of Clinical Pathology/Laboratory Medicine and the Brazilian Society of Endocrinology and Metabolism considers increased risk for intoxication when the values of 25(OH)D are above 100 ng/mL (11,38).

The usual recommended dose for correction of vitamin D deficiency [25(OH)D < 20 ng/mL] is 50,000 IU/week or 7,000 IU/day for 6-8 weeks. For maintenance, the dose varies from 400 to 2,000 IU/day, depending on the age and clinical condition of the individual (11,38) (Table 2).

**Table 2:** Recommended maintenance doses per day of cholecalciferol according to age

Age group	General population (IU)	Population at risk (IU)
0-12 months	400	400-1,000
1-8 years	400	600-1,000
9-18 years	600	600-1,000
19-70 years	600	1,500-2,000
>70 years	800	1,500-2,000
Pregnant women 14-18 years	600	600-1,000
Pregnant women > 18 years	600	1,500-2,000
Lactating women 14-18 years	600	600-1,000
Lactating women > 18 years	600	1,500-2,000

According to references: 11 and 38. (IU – international units; 1.0 IU = 0.025 mcg).

In conclusion, experimental and clinical data demonstrate that vitamin D has a physiological role in lung function and the innate and adaptive immune response against microorganisms. Cells from the respiratory tract can produce active vitamin D (calcitriol) and have its receptor in their nucleus. Vitamin D deficiency could increase the risk of acute respiratory infections and seems to impair the efficiency of gas exchanges, increasing the risk of asthma and COPD exacerbation. Current evidence has shown that individuals with low levels of 25(OH)D could experience additional benefits in terms of prevention of respiratory infections and improvement of pulmonary function after vitamin D replacement.

Due to vitamin D's peculiar metabolism, its deficiency is strongly related to low solar exposition, a very common situation during winter and spring, and especially in some populations such as the elderly, obese, and those with chronic diseases, conditions also associated with the most severe COVID-19 presentation.

Although the protective effect of vitamin D against severe COVID-19 remains speculative, there are reasons to strongly recommend vitamin D supplementation to avoid severe deficiency, especially during this pandemic, in conditions of social isolation and wintertime. Furthermore, vitamin D supplementation is usually indicated for the general population due to the clear benefits to the musculoskeletal system.

The recommended doses of vitamin D vary from 400 to 2,000 IU/day, according to different stages of life and clinical conditions. These doses are sufficient to prevent severe deficiency and are very safe, without the risk of intoxication. Therefore, they can be indicated without the need for blood measurements at this moment.
